# Inhibition of STAT3 by Anticancer Drug Bendamustine

**DOI:** 10.1371/journal.pone.0170709

**Published:** 2017-01-26

**Authors:** Kazunori Iwamoto, Yutaka Uehara, Yukie Inoue, Kyoko Taguchi, Daisuke Muraoka, Naohisa Ogo, Kenji Matsuno, Akira Asai

**Affiliations:** Center for Drug Discovery, Graduate School of Pharmaceutical Sciences, University of Shizuoka, Shizuoka, Japan; Kanazawa University, JAPAN

## Abstract

Bendamustine (BENDA), which bears the bis(2-chloroethyl)amino moiety, is an alkylating agent that stops the growth of cancer cells by binding to DNA and interfering with its replication. However, the mechanism of action underlying its excellent clinical efficacy remains unclear. In this work, we report that BENDA inhibits signal transducer and activator of transcription 3 (STAT3). In an AlphaScreen-based biochemical assay using recombinant human STAT3, binding of STAT3–Src homology 2 (SH2) to the phosphotyrosine (pTyr, pY) peptide was inhibited by BENDA but not by the inactive metabolite dihydroxy bendamustine (HP2). When a single point mutation of C550A or C712A was introduced into recombinant human STAT3, its sensitivity to BENDA was substantially reduced, suggesting that these cysteine residues are important for BENDA to inhibit STAT3. Furthermore, BENDA suppressed the function of cellular STAT3 as a transcriptional activator in a human breast cancer cell line, MDA-MB-468, with constitutively activated STAT3. A competitive pull-down assay using biotinylated BENDA (Bio-BENDA) revealed that BENDA bound tightly to cellular STAT3, presumably through covalent bonds. Therefore, our results suggest that the anticancer effects of BENDA may be associated, at least in part, with its inhibitory effect on the SH2 domain of STAT3.

## 1. Introduction

**Fig 1:**
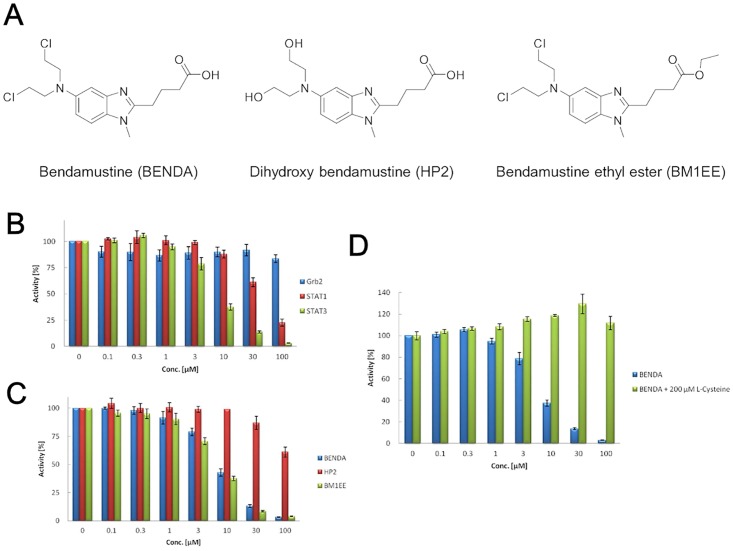
Inhibition of STAT3–SH2 binding by BENDA and its analogs. (A) Chemical structures of BENDA, HP2, and BM1EE. (B) Inhibition of SH2 binding of STAT3, STAT1, and Grb2 to the phosphotyrosine peptide by BENDA in the AlphaScreen assay. (C) Inhibitory activity of BENDA, HP2, and BM1EE against the SH2 binding of STAT3 in the AlphaScreen assay. (D) Inhibitory activity of BENDA and BENDA + 200 μM L-cysteine against the SH2 binding of STAT3 in the AlphaScreen assay. The results were obtained from three independent experiments.

Bendamustine (BENDA; 4-{5-[bis(2-chloroethyl)amino]-1-methyl-2-benzimidazolyl} butyric acid) is an alkylating agent that has clinical activity against various human cancers, including non-Hodgkin’s lymphoma [1, 2], chronic lymphocytic leukemia [3], and multiple myeloma [4, 5]. BENDA consists of a 2-chloroethylamine alkylating group, a benzimidazole ring, and a butyric acid side chain (Fig 1A). The 2-chloroethylamine alkylating group is common to other nitrogen mustard alkylators, including cyclophosphamide, chlorambucil, and melphalan, and chlorambucil also contains a butyric acid side chain. However, the benzimidazole central ring system is unique to BENDA. The benzimidazole ring structure may contribute to the unique antitumor activity of BENDA compared with conventional 2-chloroethylamine alkylating agents [6]. A major route of BENDA metabolism is hydrolysis to inactive dihydroxy BENDA (HP2), which makes little or no contribution to the anticancer effects of BENDA [7].

Similar to other alkylating agents, BENDA is a DNA cross-linking agent that causes DNA damage. However, the single-strand and double-strand DNA damage caused by BENDA is more extensive and considerably more durable than that caused by cyclophosphamide, cisplatin, and carmustine [8]. Alkylating agents mediate DNA damage that is linked to a regulated form of necrotic cell death [9]. BENDA alone [10–12] or in combination with other anticancer agents [13] also possesses proapoptotic activity in several tumor models.

The National Cancer Institute In Vitro Cell Line Screening Project suggested that BENDA may have unknown mechanisms of action that would explain its distinct pattern of cytotoxicity and unique mechanistic features compared with conventional alkylators (cyclophosphamide, chlorambucil, and melphalan) [14]. However, despite its superior anticancer activity, biochemical studies determining the mechanisms of action of BENDA have not been performed.

Signal transducer and activator of transcription (STAT) proteins play dual roles as signal transducers and transcription factors. The STAT family comprises STAT1–STAT4, STAT6, and the closely related STAT5a and STAT5b proteins. STAT proteins were first discovered as latent cytoplasmic transcription factors mediating signals from cytokine receptors and growth factor receptors to the nucleus. STAT proteins play critical roles in inflammation, proliferation, differentiation, apoptosis, survival, and immune responses [15–20]. These signaling pathways involve the activation of receptor tyrosine kinases, such as epidermal growth factor and platelet-derived growth factor receptors, and JAKs. After tyrosine residue 705 (Tyr705) is phosphorylated, two STAT monomers dimerize through a reciprocal interaction between phosphotyrosine (pTyr, pY) and the Src homology 2 (SH2) domain. The STAT3 dimers then translocate to the nucleus, where they regulate gene expression by binding to specific DNA sequences [21–24].

STAT3 is constitutively activated in many types of hematopoietic and solid tumors. For example, STAT3 is persistently tyrosine phosphorylated and constitutively activated in pancreatic, breast, lung, prostrate, ovarian, colon, gastric, and head and neck cancers, as well as in melanoma, leukemia, multiple myeloma, and lymphoma [25].

The transcription of a number of genes involved in cell cycle progression is activated by STAT3, such as cyclin D1 and c-myc, as well as genes involved in angiogenesis (e.g., VEGF) and antiapoptosis (e.g., survivin, Bcl-2, Bcl-xL). Most STAT3 target genes are key components in the regulation of cell growth, transformation, cell cycle progression, survival, metastasis, and invasion [26]. In one study, cisplatin combined with a Jak2 inhibitor (ruxolitinib) dramatically suppressed the growth of cisplatin-resistant cells with elevated JAK2 and STAT3 expression by downregulating the expression of phosphorylated STAT3 [27].

In our screening campaign for STAT3 inhibitors, we identified BENDA as a hit compound due to its inhibition of the interaction between the SH2 domain of STAT3 and the phosphor-peptide. In this study, we report the mode of STAT3 inhibition by BENDA both in cell-free system and cancer cells.

## 2. Materials and Methods

### 2.1. Reagents

Primary antibodies against STAT3, pY705 STAT3, and c-Myc were purchased from Cell Signaling Technology (Cambridge, MA, USA). Primary antibody against GAPDH was purchased from Abcam (Cambridge, MA, USA). HRP-conjugated anti-mouse IgG and anti-rabbit IgG and streptavidin-HRP conjugate were obtained from GE Healthcare Bio-Sciences (Piscataway, NJ, USA). BENDA, HP2, and BM1EE were provided by SymBio Pharmaceuticals Limited (Minato-ku, Tokyo, Japan). Peptides were synthesized by NippiBiomatrix Laboratory (Adachi-ku, Tokyo, Japan). Anti-5-carboxyfluorescein (FITC) acceptor beads and streptavidin-coated donor beads were purchased from PerkinElmer Life Sciences (Waltham, MA, USA).

### 2.2. Cell Lines and Culture

A human breast cancer cell line (MDA-MB-468) was obtained from the American Type Culture Collection (Manassas, VA, USA). Cells were grown in DMEM with 10% FBS, 20 U/mL penicillin, and 20 μg/mL streptomycin in a humidified 37°C incubator with 5% CO_2_.

### 2.3. Plasmid Construction and Protein Expression

Full-length cDNAs were obtained from a human cDNA library by PCR and cloned into pBluescript II KS (+). The nucleotides coding for human STAT3 in pBluescript II KS (+) were cloned into the HindIII/XhoI sites of modified pET-28a (+). Avi-tag and 6His-tag were introduced at the N-terminus of the proteins. *Escherichia coli* BirA-BL21 competent cells transfected with the constructed pET-28a (+) plasmid were grown at 37°C in LB medium containing 10 μg/mL chloramphenicol and 10 μg/mL kanamycin to an OD_600_ of 0.4. Cells were induced with 1 mM isopropyl-b-D-thiogalactopyranoside and 50 μM biotin for 3 h at 30°C and then harvested by centrifugation. The bacterial pellet was suspended in lysis buffer (10% sucrose, 40 mM HEPES-KOH [pH 7.8], 500 mM NaCl, 1 mM EDTA, 0.5% Nonidet P-40 [NP-40], 1 mM DTT, and 1 tab/50 mL complete protease inhibitor cocktail tablets) and lysed by 10 cycles of sonication, each consisting of a constant 20-s pulse at 0°C. Cell lysates were centrifuged at 20,000 × *g* for 15 min at 4°C, and then the supernatant was loaded onto a Ni Sepharose column. Proteins non-specifically bound to the column were washed with washing buffer (5% sucrose, 40 mM HEPES-KOH [pH 7.8], 300 mM NaCl, 1 mM EDTA, 0.6% Briji, 1 mM DTT, and 1 tab/50 mL complete protease inhibitor cocktail tablets). Recombinant proteins were eluted with elution buffer (5% sucrose, 40 mM HEPES-KOH [pH 7.8], 300 mM NaCl, 1 mM EDTA, 0.6% Briji, 1 mM DTT, and 1 tab/50 mL complete protease inhibitor cocktail tablets). Imidazole in the elution fraction was removed by dialysis against a solution containing 10 mM HEPES-KOH (pH 7.8), 50 mM NaCl, 1 mM EDTA, 0.02% (w/v) NP-40, 0.2 mM phenylmethylsulfonyl fluoride, and 10% (v/v) glycerol at 4°C overnight. Purified proteins were analyzed by SDS-PAGE and western blotting.

Point mutations were introduced into recombinant human STAT3 (rhSTAT3) by inverse PCR. We mutated Cys468, Cys542, Cys550, Cys687, and Cys712 near the SH2 domain to alanine. Inverse PCR was performed by using pBluescript II KS (+) as a template, KOD-Plus-ver. 2 (Toyobo, Osaka, Japan), and the following oligonucleotide primer set: Cys468 (forward: 5’-TCCAACATCG CTCAGATGCC AAATG-3’, reverse: 5′-GATCACCACA ACTGGCAAGG AGTGG-3′); Cys542 (forward: 5′-GAATTATTCA GGGGCTCAGA TCACATGGGC TAAAT-3′, reverse: 5′-ACACCAGGTC CCAAGAGTTT CTCTGCC-3′); Cys550 (forward: 5′-GCTAAATTTG CCAAAGAAAA CATGGCTGGC-3′, reverse: 5′-CCATGTGATC TGACACCCTG AATAATTCA CACC-3′); Cys687 (forward: 5′-GGAAAGTATG CTCGGCCAGA GAGCCAG-3′, reverse: 5′-GAATGCCTCC TCCTTGGGAA TGTCAGG-3′); and Cys712 (forward: 5′-AAGTTTATCG CTGTGACACC AACGACCTGC-3′, reverse: 5′-GGTCTTCAGG TATGGGGCAG CGCTAC-3′). Inverse PCR (1 × PCR buffer, 1.5 mM MgSO4, 15 pmol 5′ primer and 3′ primer, 0.2 mM dNTPs, 50 ng STAT3 cDNA, 1 U KOD Plus) was performed under the following conditions: 2 min at 94°C, (10 s at 98°C, and 6.5 min at 68°C)×7 cycles. The amplified fragment was ligated by using Ligation High ver. 2 (Toyobo). The resultant plasmid was cloned into the HindIII/XhoI sites of modified pET-28a (+). The nucleotides coding for human mutant STAT3 in pET-28a (+) were named mutant STAT3 His-Avi-pET28a (+).

### 2.4. Alpha Screen Assays

AlphaScreen assays [28] were performed in a final reaction volume of 25 μL of the assay buffer, containing 10 mM HEPES-NaOH (pH 7.4), 50 mM NaCl, 1 mM EDTA (pH 8.0), 0.1% NP-40, and 10 ng/mL BSA in a 96-well microtiter plate at 25°C. The labeled Phospho-Tyr (pTyr) peptide probe was 5-carboxyfluorescein (FITC)-GpYLPQTV for STAT3, FITC-GpYDKPHVL for STAT1, and FITC-PSpYVNVQN for Grb2. A DMSO solution of BENDA was incubated with 50 nM of each SH2-containing protein for 15 min, 50 nM of the corresponding FITC-pTyr peptide was added, and the mixture was incubated for 90 min. Streptavidin-coated donor beads and anti-FITC acceptor beads were added and incubated for 90 min before the signals were measured with an Envision Xcite plate reader (PerkinElmer Life Sciences, Waltham, MA, USA).

### 2.5. Immunoprecipitation

MDA-MB-468 cells were treated with biotinylated BENDA (Bio-BENDA) in the presence or absence of an excess amount of free drugs for 4 h and then lysed in PhosphoSafe™ Extraction Reagent (Merck, Darmstadt, Germany). Lysate (200 μL) was precleared with a mixture of Protein G Plus/Protein A Agarose Suspension (EMD Millipore, Billerica, MA, USA) for 1 h at 4°C and protein A and G agarose were removed by centrifugation at 13,000 rpm for 10 min. The lysate (200 μL) was immunoprecipitated with STAT3 antibody (1 μL) overnight at 4°C on a shaker and the immunocomplex was captured by the addition of a Protein G Plus/Protein A Agarose Suspension (30 μL) slurry for 1 h at 4°C. Samples were washed five times with lysis buffer and then boiled four times in SDS-PAGE sample buffer and run on an SDS-PAGE gel. The protein was transferred to a nitrocellulose membrane. The membranes were blocked in 5% skim milk in TBS/0.1% Tween 20. They were then incubated with streptavidin-HRP diluted at 1:2000 in 1.5% skim milk in TBS (pH7.4)/0.1% Tween 20 overnight at 4°C. Immunoblots were developed by using a chemiluminescent substrate.

### 2.6. Competition Assay with Cysteine-blocking Reagent

rhSTAT3 (0.89 μM) in 100 mM HEPES was incubated with 10 μM Alexa Fluor 488 C5 maleimide (Invitrogen, Paisley, UK), a thiol-reactive probe, in the presence or absence of 50 or 100 μM BENDA or HP2 for 2 h at room temperature. Samples were diluted in SDS-PAGE sample buffer without boiling or 2-mercaptoethanol and run on an SDS-PAGE gel. The protein was transferred to a nitrocellulose membrane and then blotted with Ni-HRP. The Alexa-labeled rhSTAT3 was analyzed by fluorescence (excitation/emission: 493/516 nm) and western blotting with Ni-HRP.

### 2.7. SH2 Binding Assay with Mutant Recombinant STAT3

Mutant recombinant STAT3, which possessed a single mutation at Cys468, Cys542, Cys550, Cys687, or Cys712, was prepared and used for the AlphaScreen assays. The 375 nM mutant recombinant STAT3 was incubated with 100 μM BENDA dissolved in DMSO for 30 min, and the AlphaScreen assays were performed.

### 2.8. Synthesis of Bio-BENDA

The synthesis and preparation of Bio-BENDA are described in Supporting Information (S1 Text with S1 Fig).

### 2.9. DNA Binding Assay

The DNA-binding activity of STAT3 was evaluated with a TransAM ELISA kit (Active Motif, Carlsbad, CA, USA). The assay and nuclear extract preparation were performed according to the manufacturer’s instructions. Heat-denatured nuclear extract was used as a negative control. The nuclear extract (20 μg) was added to each well of a 96-well microtiter plate coated with immobilized oligonucleotides containing the STAT3 consensus binding site and incubated at room temperature for 1 h. They were incubated with anti-STAT3 antibody for 1 h at room temperature, followed by HRP-conjugated antibody for 1 h at room temperature. The developing solution was added, the reaction was terminated by the addition of stop solution, and the absorbance was measured at 450 nm with a reference wavelength of 655 nm (SpectraMax M5SK, Molecular Devices, Sunnyvale, CA, USA).

### 2.10. Fluorescence Resonance Energy Transfer (FRET) Assay

The expression vectors for the fusion proteins STAT3-CFP and STAT3-YFP were prepared by the addition of STAT3 cDNA to pAmCyan1-N1 and pZsyellow1-N1. The vector mixture (1:1) was transfected into HEK293 cells with Lipofectamine 2000. The cells were cultured with 2% FBS DMEM for 48 h in a 96-well microtiter plate, treated with 0, 10, 20, or 50 μM BENDA or HP2 in a final DMSO concentration of 0.2% for 24 h, and then stimulated with 50 ng/mL IL-6 for a further 1 h. Fluorescence signals were measured (SpectraMax M5SK). The signals measured by FRET (excitation/emission: 436/528 nm) were corrected for crosstalk signals from cyan (excitation/emission: 436/488 nm) and yellow (excitation/emission: 517/528 nm).

### 2.11. ChIP Assay

A ChIP assay was performed with an ExactaChIP Human/Mouse STAT3 ChIP kit (R&D Systems, Minneapolis, MN, USA). MDA-MB-468 cancer cells were fixed with 1% formaldehyde and treated with the lysis buffer. After sonication, anti-STAT3 antibody or normal mouse IgG was added to the extract, followed by precipitation with Protein G Plus/Protein A Agarose Suspension. The eluted DNA fraction was used for PCR with human c-myc primers.

### 2.12. RT-PCR

MDA-MB-468 cancer cells were treated with 0, 50, or 100 μM BENDA for 8 h. Total RNA was extracted with RNeasy kits (Qiagen, Valencia, CA, USA) and reverse-transcribed to cDNA with the One-Step SYBR PrimeScript RT-PCR Kit II (Takara bio, Shiga, Japan). PCR amplification was performed under the following conditions: 5 min at 42°C, 10 s at 95°C, 40 cycles of 30 s at 94°C, 30 s at 55°C and 30 s at 72°C, and a final 5-min extension at 72°C. The DNA sequences of the primers for the STAT3 downstream target genes used for RT-PCR analysis were c-myc (forward: 5’-TACCCTCTCA ACGACAGCAG-3’, reverse: 5’-TCTTGACATT CTCCTCGGTG-3’) [29], cyclin D1 (forward: 5’-GCTGGAGCCC GTGAAAAAGA-3’, reverse: 5’-CTCCGCCTCT GGCATTTTG-3’), survivin (forward: 5’-ACCAGGTGAG AAGTGAGGGA-3’, reverse: 5’-AACAGTAGAG GAGCCAGGGA-3’), Bcl-2 (forward: 5’-TCTTTGAGTT CGGTGGGGTC-3’, reverse: 5’-TGCATATTTG TTTGGGGCAGG-3’), and GAPDH (forward: 5’-TGATGACATC AAGAAGGTGG TGAAG-3’, reverse: 5’-TCCTTGGAGG CCATGTGGGC AT-3’) [30]. The mRNA expression levels of the STAT3 target genes in cells treated with BENDA or HP2 were evaluated by quantitative RT-PCR (PikoReal, Thermo Fisher Scientific, Waltham, MA, USA) according to the manufacturer’s protocol. Primers were obtained from Sigma-Aldrich (St. Louis, MO, USA).

### 2.13. Western Blotting

Cells were treated with PhosphoSafe Extraction Reagent. Total proteins (40 μg) were subjected to SDS-PAGE and then transferred onto a nitrocellulose membrane. Membranes were blocked in 5% skim milk in TBS/0.1% Tween 20, incubated with primary antibodies diluted at 1:1000 in 1.5% skim milk in TBS (pH 7.4)/0.1% Tween 20 overnight at 4°C, and then incubated with HRP-conjugated secondary antibodies diluted at 1:5000 in TBS (pH 7.4)/0.1% Tween 20 for 1 h at room temperature. Immunoblots were developed with a chemiluminescent substrate.

### 2.14. WST Cell Proliferation Assays

Cells were plated in a 96-well tissue culture plate (1000 cells per well) and incubated for 48 h. After incubation, the cells were treated with vehicle, 0.1–100 μM BENDA, or HP2 for 48 h. Samples were assayed in triplicate. After incubation, WST-8 solution (10 μL; Dojindo Molecular Technologies, Rockville, MD, USA) was added to each well and incubated at 37°C for 4 h, and the absorbance was read at 450 nm.

### 2.15. Statistical Analysis

All experiments were performed in triplicate. In the AlphaScreen assay, the statistical significance was calculated by the Student’s *t*-test with *P* < 0.01 versus wild-type rhSTAT3. For RT-PCR and the FRET and DNA binding assays, the statistical significance was calculated by the Student’s *t*-test with *P* < 0.05 and *P* < 0.01 versus vehicle treatment.

## 3. Results

### 3.1. Identification of BENDA as an Antagonist of STAT3–SH2 in Vitro

We have previously identified several STAT3–SH2 selective antagonists, such as diphenylporphyrin, quinolinecarboxamide, and 1,4-naphthalenedione derivatives, by using AlphaScreen SH2 binding assays [28, 31, 32]. In our continuous screening campaign using the AlphaScreen assay, we identified BENDA (Fig 1A) as another hit compound due to its concentration-dependent inhibition of the interaction of the SH2 domain of rhSTAT3 with the phosphor-peptide (Fig 1B). BENDA selectively antagonized STAT3–SH2 (IC_50_ = 7.4 μM) in vitro over the other SH2-containing proteins STAT1 (IC_50_ = 60 μM) and Grb2 (IC_50_ > 100 μM) (Fig 1B). The chemical structures of BENDA, HP2, and BENDA ethyl ester (BM1EE) are shown in Fig 1A. HP2, an inactive metabolite of BENDA, did not inhibit STAT3–SH2 binding (IC_50_ > 100 μM). In contrast to HP2, BM1EE inhibited STAT3–SH2 binding (IC_50_ = 6.1 μM) with a similar potency to BENDA (Fig 1C). However, the SH2 inhibitory activity of BENDA was attenuated by the addition of excess L-cysteine (200 μM) to the assay mixture (Fig 1D). In addition, BENDA did not inhibit the SH2 binding in the presence of 200 μM of 2-mercaptoethanol (data not shown). These results show that the thiol group could inactivate BENDA through a nucleophilic attack at the 2-chloroethylamine alkylating group, suggesting that BENDA may covalently bind to cysteine residues in STAT3.

### 3.2. BENDA Binds to Cysteine Residues at Positions Cys550 and Cys712 in STAT3

**Fig 2:**
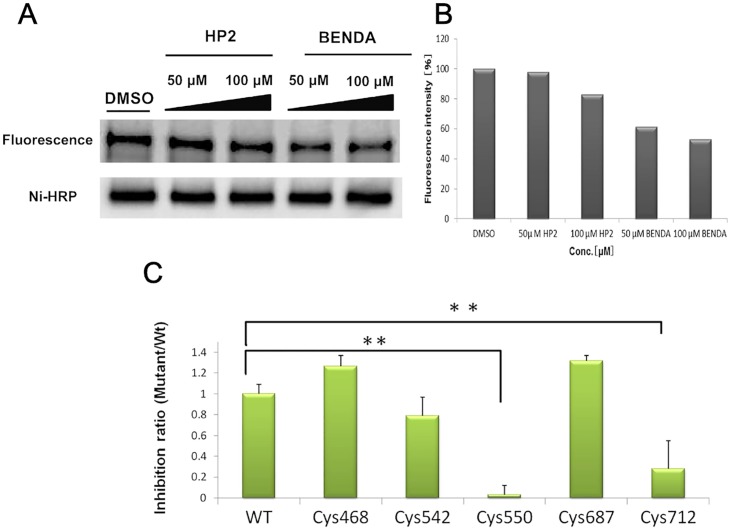
Cysteine-mediated binding of BENDA to STAT3–SH2. (A) Competitive inhibition of the STAT3–SH2 binding with a thiol-reactive probe. rhSTAT3 was incubated with Alexa Fluor 488 C5 maleimide in the presence or absence of BENDA or HP2 for 2 h. Incorporation of Alexa into rhSTAT3 was analyzed by fluorescence (excitation/emission: 493/516 nm). The total level of His-tagged STAT3 protein was analyzed by Ni-HRP. (B) Quantification of Alexa-labeled STAT3 fluorescence intensity levels. (C) Sensitivity of single-mutated STAT3 proteins (Cys468, Cys542, Cys550, Cys687, and Cys712) to BENDA. SH2 binding to the phosphotyrosine peptide was assessed by the AlphaScreen assay. The means ± SD of three separate experiments are plotted (**P < 0.01 versus wild-type rhSTAT3; Student’s *t*-test).

We examined whether BENDA bound to STAT3 at cysteine residues. rhSTAT3 was incubated with Alexa Fluor 488 C5 maleimide, a nonspecific thiol-reactive probe, in the presence or absence of BENDA or HP2 for 2 h. BENDA was added first and then the Alexa conjugated STAT3 was made in this assay conditions. The Alexa-labeled rhSTAT3 was analyzed by fluorescence (excitation/emission: 493/516 nm) and western blotting with Ni-HRP. The fluorescence intensity of the Alexa-labeled rhSTAT3 was inhibited by BENDA in a dose-dependent manner (S2 Fig). The fluorescence intensity was reduced to 62% and 52% in the presence of 50 and 100 μM BENDA, respectively but the effect was hardly observed in the presence of HP2 (Fig 2A and 2B). This competitive inhibition of the thiol-reactive reagent by BENDA, but not HP2, suggested that 2-chloroethylamine alkylating moiety of BENDA blocked thiol groups on the rhSTAT3 protein.

To determine which cysteine residues are blocked by BENDA, we assessed its inhibitory activity against point mutant recombinant STAT3 proteins, which possessed an alanine mutation at Cys468, Cys542, Cys550, Cys687, or Cys712. These mutant proteins were prepared and used in the AlphaScreen SH2 binding assay. The Cys550 or Cys712 mutants showed inhibitions of 3% and 25%, respectively, which were significantly lower than that of wild-type rhSTAT3 (Fig 2C) at 100 μM BENDA. These results suggest that BENDA covalently bound to cysteine residues at positions Cys550 and Cys712 and thereby inhibited the binding of SH2 to the corresponding p-Tyr peptide.

### 3.3. BENDA Selectively Binds to Cellular STAT3

**Fig 3:**
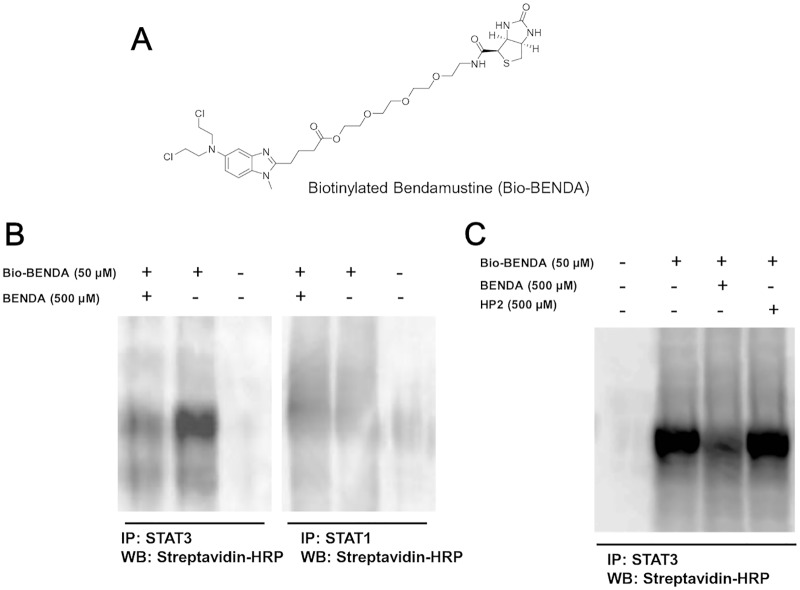
Pull-down assay using biotinylated BENDA. (A) Chemical structure of Bio-BENDA. (B) Pull-down assay using Bio-BENDA. MDA-MB-468 cells were treated with Bio-BENDA in the presence or absence of excess free BENDA for 4 h. Cellular STAT3 was immunoprecipitated with anti-STAT3 or anti-STAT1 antibody and Bio-BENDA binding to STAT3 was detected with streptavidin-HRP. (C) Pull-down assay using Bio-BENDA. MDA-MB-468 cells were treated with Bio-BENDA in the presence or absence of excess free BENDA or HP2 for 4 h. Cellular STAT3 was immunoprecipitated with anti-STAT3 antibody and Bio-BENDA binding to STAT3 was detected with streptavidin-HRP.

To investigate whether BENDA bound to cellular STAT3, we designed and synthesized Bio-BENDA (Fig 3A). The comparable potency of BM1EE to that of BENDA (Fig 1C) led us to use the carboxylic acid in conjunction with biotin. To reduce steric hindrance, a spacer with 13 atoms was expected to be suitable for the interaction with streptavidin [33]. MDA-MB-468 cells were treated with Bio-BENDA in the presence or absence of BENDA or HP2 for 4 h. First, the cellular STAT3 was immunoprecipitated with anti-STAT3 or anti-STAT1 antibody and detected by immunoblotting with streptavidin-HRP. After immunoprecipitation of cellular STAT3 with anti-STAT3 antibody, no band was observed by blotting with streptavidin-HRP. However, when the cells were treated with 50 μM Bio-BENDA, the STAT3 band was observed by streptavidin-HRP, and the band intensity was competitively reduced by the addition of excess free BENDA. In contrast to STAT3, the immunoprecipitation of cellular STAT1 with the anti-STAT1 antibody from the cells treated with 50 μM Bio-BENDA resulted in no band corresponding to STAT1. (Fig 3B). Furthermore, no reduction of the STAT3 band intensity was observed with HP2, in contrast to BENDA (Fig 3C). These results suggest that BENDA selectively bound to cellular STAT3 over STAT1 by using the 2-chloroethylamine alkylating moiety.

### 3.4. BENDA Inhibits STAT3 Dimerization and the DNA-binding Activity of STAT3

**Fig 4:**
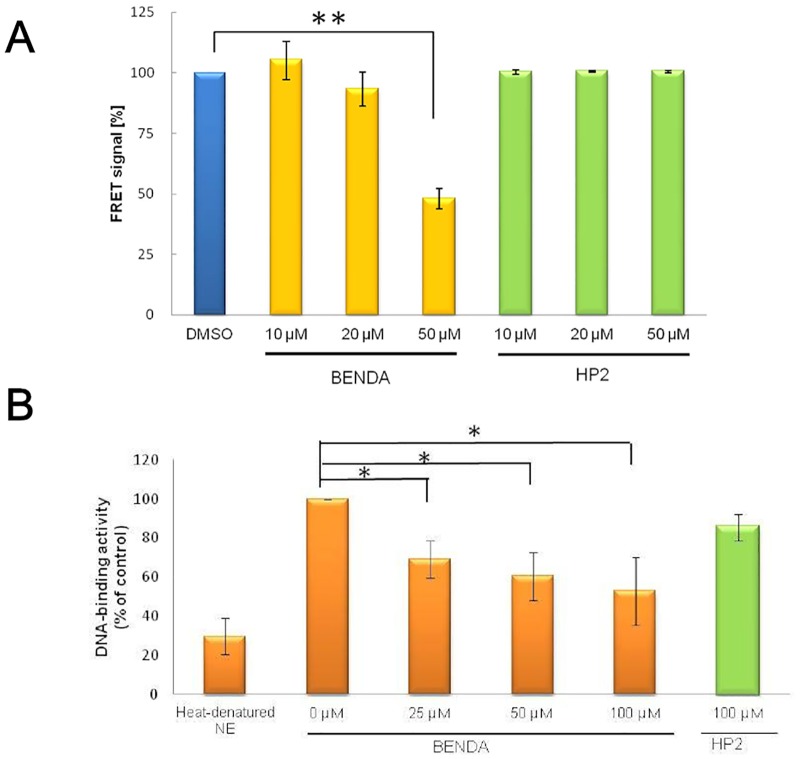
Inhibition of STAT3 dimerization and DNA binding by BENDA. (A) Effect of BENDA on FRET signals generated by interactions between STAT3-CFP and STAT3-YFP in HEK293 cells. The transient transfectant was treated with the indicated concentrations of BENDA for 23 h after stimulation with IL-6 for 1 h (**P < 0.01 versus vehicle; Student’s *t*-test). (B) Effect of BENDA on the DNA-binding activity of nuclear STAT3. MDA-MB-468 cells were treated with BENDA or HP2 for 24 h. Heat-denatured nuclear extract (NE) was used as a negative control. DNA-binding activity of STAT3 was evaluated by using a TransAM ELISA kit (*P < 0.05 versus blank; Student’s *t*-test). The results were obtained from three independent experiments.

To evaluate BENDA inhibition of STAT3 dimerization in cells, a FRET assay was performed. HEK293 cells were co-transfected with STAT3-CFP and STAT3-YFP. IL-6 stimulation induced a high FRET signal due to the intracellular interaction between STAT3-CFP and STAT3-YFP. Pretreatment of the cells with BENDA before IL-6 stimulation reduced the FRET signals. In contrast, the FRET signals were unchanged when the cells were treated with HP2 (Fig 4A).

We examined whether BENDA suppressed both the DNA-binding and transcriptional activity of STAT3 in human breast cancer cells expressing activated STAT3. An ELISA assay with the consensus binding site of the oligonucleotides immobilized in a 96-well microtiter plate was used to analyze the nuclear lysates from MDA-MB-468 cells treated with 0, 25, 50, or 100 μM BENDA or 100 μM HP2 for 24 h. BENDA inhibited binding of STAT3 against oligonucleotides containing a STAT3 consensus binding site in a concentration-dependent manner but HP2 did not. (Fig 4B).

### 3.5. BENDA Decreases the Expression of c-myc, Which is Transcriptionally Regulated by STAT3

**Fig 5:**
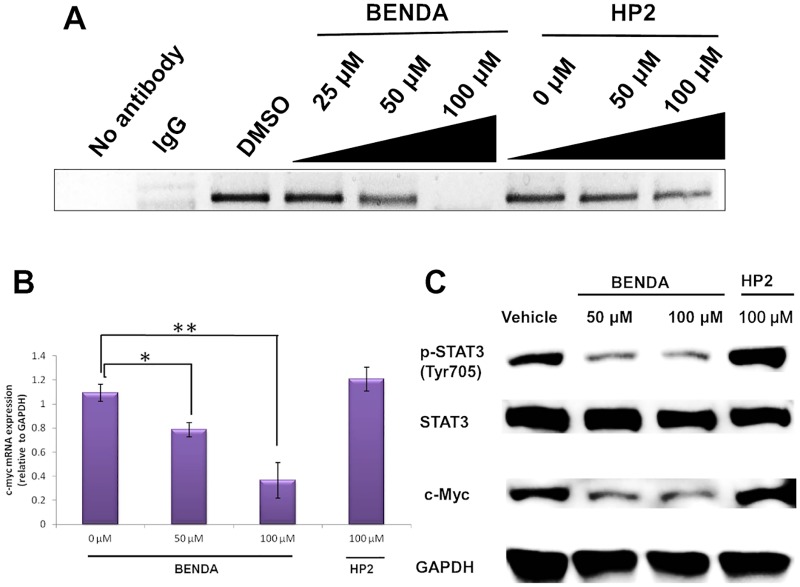
Inhibition of STAT3 function in cancer cells by BENDA. (A) Effect of BENDA on c-myc promoter binding by STAT3. MDA-MB-468 cells were treated with BENDA or HP2 for 24 h. The ChIP assay was performed using an ExactaChIP Human/Mouse STAT3 ChIP kit. IgG was used as a negative control. (B) Effect of BENDA on c-myc mRNA expression. MDA-MB-468 cells were treated with the indicated concentrations of BENDA or HP2 for 8 h. The mRNA expression levels of c-myc were evaluated by RT-PCR. The means ± SD of three separate experiments are plotted (*P < 0.05 and **P < 0.01 versus vehicle; Student’s *t*-test). (C) Effect of BENDA on Tyr705 phosphorylation of STAT3 and the expressions of STAT3 and c-Myc protein. MDA-MB-468 cells were treated with the indicated concentrations of BENDA or HP2 for 24 h and lysed for western blotting analysis.

To elucidate the effect of BENDA on the STAT3–DNA complex in MDA-MB-468 cells expressing persistently activated STAT3, a ChIP assay was performed. MDA-MB-468 cells were treated with 0, 25, 50, or 100 μM BENDA or 0, 50, or 100 μM HP2 for 8 h. Protein—DNA complexes were fixed with 1% formaldehyde and immunoprecipitated with an anti-STAT3 antibody. The complex was subjected to PCR amplification for the detection of the c-myc promoter, which is a target for transcriptional activation of STAT3 [26]. The amplified c-myc promoter was clearly detected in the presence of HP2, whereas the band intensity was reduced in the presence of BENDA (Fig 5A).

The mRNA expression levels of c-myc were evaluated by RT-PCR. MDA-MB-468 cells were treated with 0, 50, or 100 μM BENDA or 100 μM HP2 for 8 h. BENDA induced a dose-dependent decrease in c-myc expression in MDA-MB-468 cells (Fig 5B). When the cells were pretreated with 50 and 100 μM BENDA, the c-myc mRNA expression was reduced to 72% and 33% of that of the vehicle treatment, respectively. These results show that BENDA blocks the DNA binding that is required for the transcriptional activity of STAT3 (Fig 5B).

We examined whether the ability of STAT3 to regulate the expression of its target genes is affected by BENDA. Using western blotting, we confirmed that BENDA inhibits the phosphorylation of STAT3-Y705. MDA-MB-468 cells were treated with vehicle, 50 or 100 μM BENDA, or 100 μM HP2, and the proteins were analyzed by western blotting. BENDA inhibited the phosphorylation of STAT3-Y705 in a concentration-dependent manner (Fig 5C). Fig 5C also shows that BENDA inhibited the expression of c-Myc protein.

### 3.6. BENDA Decreases the Expression of STAT3 Target Genes

**Fig 6:**
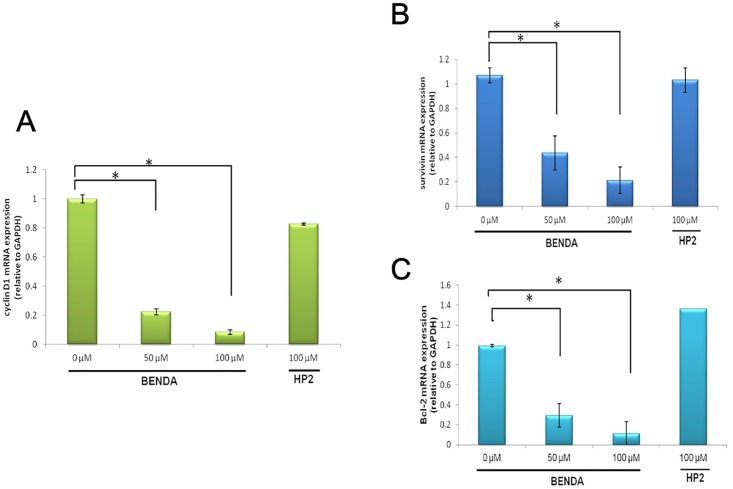
Effect of BENDA on the expression of STAT3 target genes. MDA-MB-468 cells were treated with the indicated concentrations of BENDA or HP2 for 8 h. The mRNA expression levels of (A) cyclin D1, (B) survivin, and (C) Bcl-2 were evaluated by RT-PCR. The means ± SD of three separate experiments are plotted (*P < 0.05 versus vehicle; Student’s *t*-test).

STAT3 activates the transcription of several genes involved in cell cycle progression, such as cyclin D1, and in antiapoptosis, such as survivin and Bcl-2 [26]. MDA-MB-468 cells were treated with 0, 50, or 100 μM BENDA or 100 μM HP2 for 8 h. The mRNA expression levels of cyclin D1, survivin, and Bcl-2 were evaluated by RT-PCR. BENDA induced a dose-dependent decrease in cyclin D1, survivin, and Bcl-2 expression in MDA-MB-468 cells (Fig 6). These results show that BENDA inhibits mRNA expression of cyclin D1, survivin, and Bcl-2 by blocking the DNA binding of STAT3 in cells.

### 3.7. BENDA Selectively Inhibits Proliferation in Human Cancers with High Levels of P-STAT3

**Fig 7:**
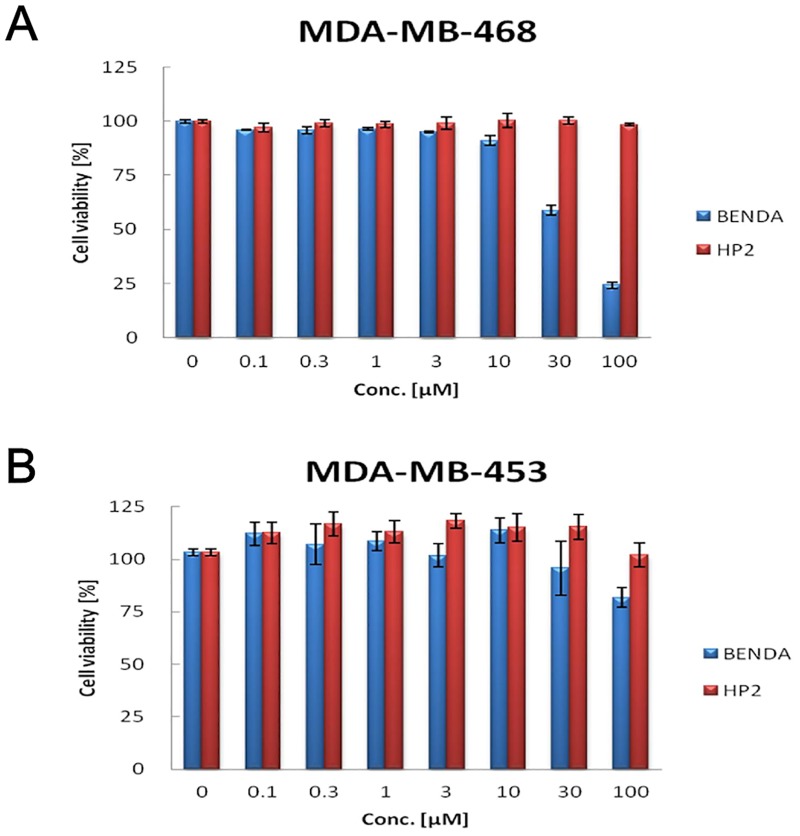
BENDA selectively inhibits proliferation in human cancers that harbor high levels of P-STAT3. (A) MDA-MB-468 cells (high P-STAT3) and (B) MDA-MB-453 cells (low P-STAT3) were plated in 96-well plates and treated with BENDA or HP2 for 48 h and processed for WST assays as described in the Materials and Methods section. The results were obtained from three independent experiments.

We investigated whether BENDA selectively inhibits proliferation in human cancers with high levels of P-STAT3. MDA-MB-468 cells were treated with 0.1–100 μM BENDA or HP2 for 48 h and analyzed by a WST cell proliferation assay. BENDA inhibited proliferation of MDA-MB-468 cells (high P-STAT3) (Fig 7A) in a dose-dependent manner, but not MDA-MB-453 cells (low P-STAT3) (Fig 7B). HP2 did not inhibit proliferation of MDA-MB-468 cells or MDA-MB-453 cells (Fig 7A and 7B). These results also indicate that BENDA mediates its effects through STAT3 inhibition.

## 4. Discussion

BENDA is widely known as a DNA alkylating agent. However, BENDA displays a distinct pattern of cytotoxicity and unique mechanistic features compared with other alkylating agents [14] and the details of the mechanism of action underlying its excellent anticancer activity largely remain unclear. In this study, we showed that a potential target protein of BENDA could be STAT3.

Initially, BENDA was unexpectedly identified as a hit compound due to its ability to antagonize STAT3–SH2 binding in our screening campaign using a previously reported AlphaScreen assay [28]. In this cell-free biochemical assay, BENDA selectively inhibited the SH2 binding of STAT3 over that of STAT1 or Grb2. The reduced inhibitory activity of the metabolite HP2 and loss of the inhibition by BENDA in the presence of an excess amount of thiol groups indicated that the bis(2-chloroethyl)amino moiety is important for the STAT3 inhibitory activity and is inactivated by the addition of thiol-containing reagent. BENDA partially competed with a nonspecific thiol-blocking reagent, Alexa Fluor 488 C5 maleimide. This partial effect suggests that BENDA binds to cysteine residues and has a more selective effect on the cysteine residues in rhSTAT3 than the maleimide derivative. We assumed that BENDA bound to cysteine residues around the SH2 domain and thus mutated Cys468, Cys542, Cys550, Cys687, or Cys712 near the SH2 domain to alanine. The sensitivity to BENDA decreased for the Cys550 and Cys712 mutants, indicating that these cysteine residues could be at least some of the candidate covalent binding sites of BENDA in STAT3. From these results, we would like to suggest that BENDA inhibits SH2 binding of STAT3 due to its covalent binding to cysteine residues including Cys550 and Cys712 around the SH2 domain in STAT3. The lower inhibitory activity of BENDA to STAT1 might be explained by modification of these cysteine residues because STAT1 does not contain these cysteine residues around the SH2 domain [15].

As a mechanism for STAT3 inhibition by BENDA, covalent binding to STAT3 through the cysteine residues around the SH2 domain is plausible, because several thiol modifiers have been reported to inhibit STAT3 [34, 35, 36, 37]. One of them, Stattic, has been reported to bind to Cys251 (coiled-coil domain), Cys259 (coiled-coil domain), Cys367 (DNA-binding domain), and Cys426 (DNA-binding domain), inhibiting STAT3 dimerization, STAT3 phosphorylation, and STAT3 binding to DNA [35]. Galiellalactone binds to Cys367 (DNA-binding domain), Cys468 (DNA-binding domain), and Cys542 (linker domain) and inhibits STAT3 binding to DNA [36]. Cys550 (linker domain) and Cys712 (transcription activation domain) are the candidate binding sites of BENDA. The binding domains of BENDA, therefore, appear to be different from those of known STAT3 inhibitors, presumably due to its bifunctional alkylating moiety.

Taken together, there could be several critical cysteine residues for the cellular function of STAT3, and BENDA might inhibit STAT3 function by binding to such cysteine residues around the SH2 domain. More detailed analysis is necessary to identify the binding domains of BENDA.

In this cultured cell system, we showed that BENDA bound to cellular STAT3 and inhibited its function in cells. The competitive pull-down assay with Bio-BENDA that was newly designed and synthesized to investigate the interaction between BENDA and cellular STAT3 indicated that BENDA tightly bound to STAT3 but not STAT1 in cancer cells. Further analysis using free BENDA suggested that BENDA inhibited STAT3 homodimerization, DNA binding, transcriptional activation, and subsequent expression of STAT3-target genes such as c-myc, survivin, cyclin D, and Bcl-2. Western blot analysis showed that phosphorylation of Tyr705 was downregulated in the cells treated with BENDA. Such downregulation has been reported in the case of other STAT3–SH2 antagonists such as Stattic [35] and S3I-201 [37]. The decrease in the phosphor-Tyr status might be caused by the suppression of the recruitment of STAT3 to Tyr kinases such as JAK and Src [38]. Alternatively, inhibition of the upstream signaling of STAT3 activation might still be a mechanism of STAT3 inhibition by BENDA in cells. The MDA-MB-468 cells possess relatively high levels of the activated STAT3 and are STAT3-dependent. Indeed the known STAT3 inhibitors such as Stattic and Eriocalyxin B are reported to inhibit the growth of MDA-MB-468 cells more than that of the MDA-MB-453 cells [39]. In our growth inhibition assay, MDA-MB-468 cells were more susceptible to BENDA than MDA-MB-453 cells. The results suggest that STAT3 inhibition by BENDA at least partly contributes to its cell growth inhibition. It is noteworthy that 50 μM BENDA transiently arrested cells in G2, while 200 μM BENDA arrested cells in S phase, suggesting a concentration-dependent difference in DNA repair efficiency [40]. The STAT3 inhibition by BENDA may relate to the concentration-dependent effect of BENDA on cell cycle and DNA repair.

BENDA (IC_50_ = 7.4 μM) inhibits STAT3–SH2 binding in the AlphaScreen assay, whereas a more than 10-fold higher concentration of BENDA was required to inhibit cellular STAT3 and growth in STAT3-activated cells. The physicochemical properties, such as stability and solubility, of BENDA possibly influenced such differences [41]. As shown in Fig 1D, BENDA was wholly inactivated in the presence of 0.2 mM cysteine or 2-mercaptoethanol in cell-free systems. Cellular thiol sources such as glutathione may also inactivate BENDA in cancer cells. Such a fragile property of BENDA would increase its effective concentration in experimental cultured cell systems.

Here, we identified STAT3 as a potential target of the anticancer drug BENDA. BENDA is a clinically useful drug for the treatment of non-Hodgkin’s lymphoma and is distinct from other alkylating agents that contain a 2-chloroethylamine alkylating group. Although further analysis is needed to elucidate the detailed mechanism of action of BENDA, our results suggest that the superior anticancer effects of BENDA may be associated, at least in part, with its inhibitory effect on the SH2 domain of STAT3.

## Supporting Information

S1 Fig Synthetic scheme of Bio-BENDA. **Reagents and conditions.** (a) TsCl, Et_3_N, CH_2_Cl_2_, rt; (b) NaN_3_, DMF, rt; (c) PPh_3_, THF, H_2_O, rt; (d) (+)-biotine, HBTU, *i*Pr_2_EtN, CH_2_Cl_2_, rt; (e) H_2_, 10% Pd-C, AcOH, H_2_O, rt; (f) BENDA HCl, *i*Pr_2_N = C = N-*i*Pr_2_, 4-DMAP, CH_2_Cl_2_, rt.
(TIF)

S2 Fig Cysteine-mediated binding of BENDA to STAT3–SH2. (A) Competitive inhibition of the STAT3–SH2 binding with a thiol-reactive probe. rhSTAT3 (0.89 μM) was incubated with 10 μM Alexa Fluor 488 C5 maleimide in the presence or absence of BENDA for 2 h. Incorporation of Alexa into rhSTAT3 was analyzed by fluorescence (excitation/emission: 493/516 nm). (B) Quantification of Alexa-labeled STAT3 fluorescence intensity levels.
(TIF)

S1 Text Synthesis of Bio-BENDA. (DOCX)
