# Supplementary figures and images for: Inhibition of STAT3 by Anticancer Drug Bendamustine
Source: PLoS One. 2017 Jan 26;12(1):e0170709. doi: 10.1371/journal.pone.0170709 (PMC5268383; doi:10.1371/journal.pone.0170709)

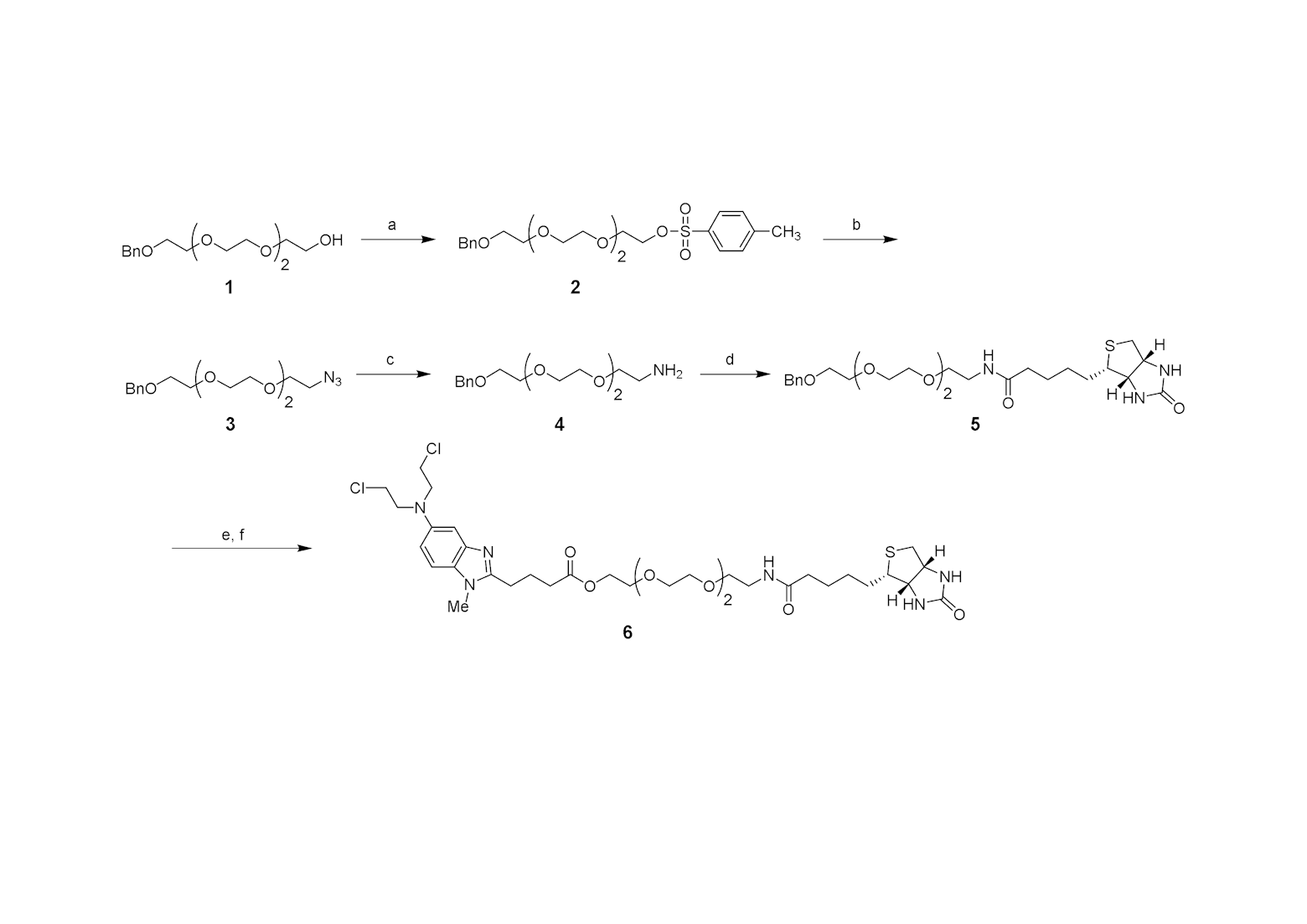

Supplement: S1 Fig — Reagents and conditions. (a) TsCl, Et3N, CH2Cl2, rt; (b) NaN3, DMF, rt; (c) PPh3, THF, H2O, rt; (d) (+)-biotine, HBTU, iPr2EtN, CH2Cl2, rt; (e) H2, 10% Pd-C, AcOH, H2O, rt; (f) BENDA HCl, iPr2N = C = N-iPr2, 4-DMAP, CH2Cl2, rt. (TIF) [file pone.0170709.s001.tif]

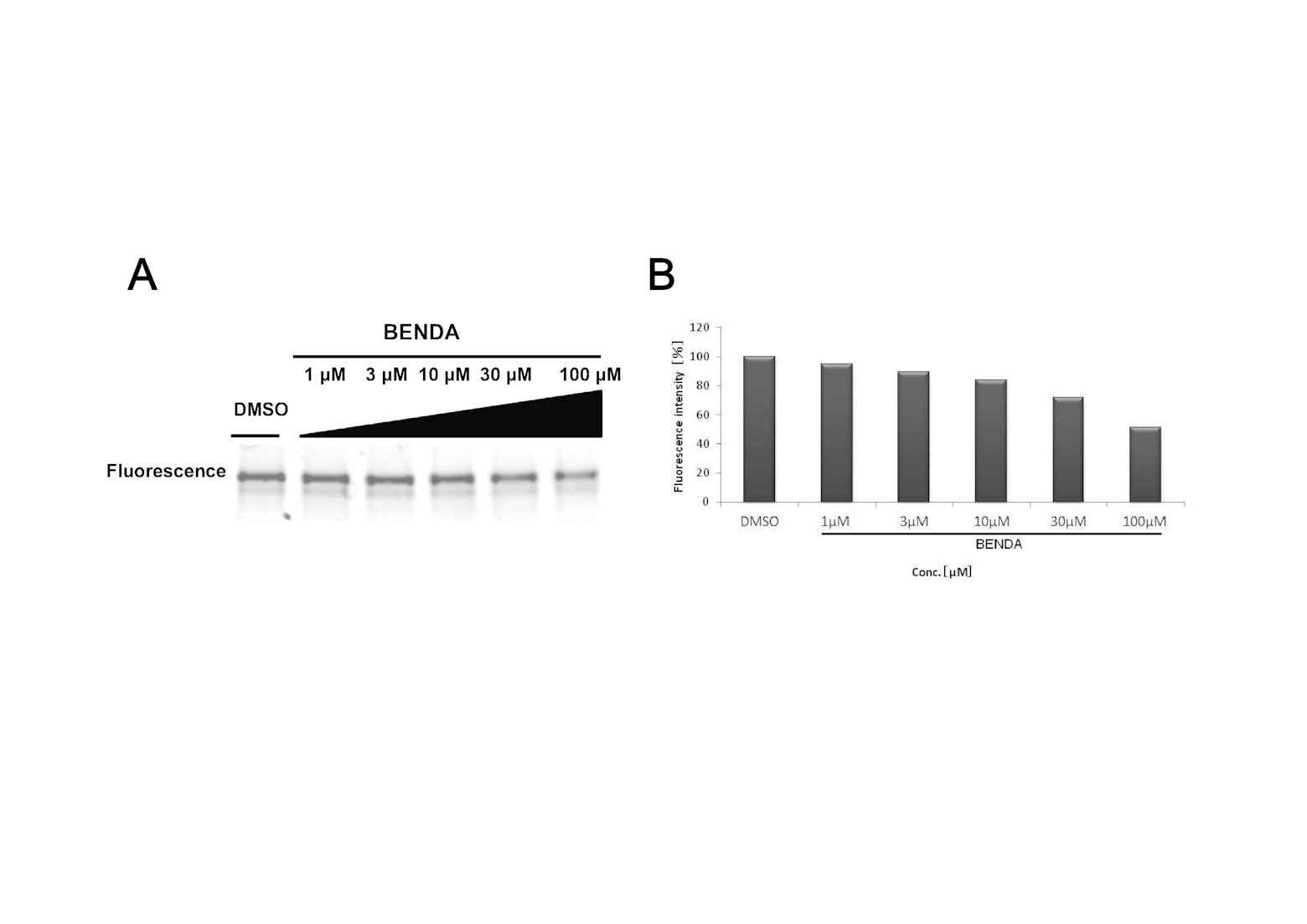

Supplement: S2 Fig — (A) Competitive inhibition of the STAT3–SH2 binding with a thiol-reactive probe. rhSTAT3 (0.89 μM) was incubated with 10 μM Alexa Fluor 488 C5 maleimide in the presence or absence of BENDA for 2 h. Incorporation of Alexa into rhSTAT3 was analyzed by fluorescence (excitation/emission: 493/516 nm). (B) Quantification of Alexa-labeled STAT3 fluorescence intensity levels. (TIF) [file pone.0170709.s002.tif]
