# Supplementary material for: Inhibition of STAT3 by Anticancer Drug Bendamustine
Source: PLoS One. 2017 Jan 26;12(1):e0170709. doi: 10.1371/journal.pone.0170709 (PMC5268383; doi:10.1371/journal.pone.0170709)
Supplement: S1 Text — (DOCX) [file pone.0170709.s003.docx]

*Synthesis of Bio-BENDA*

*General Methods*

^1^H NMR spectra were recorded in the indicated solvent (ECA-500 or A-500, JEOL, Tokyo). Chemical shifts (δ) are expressed in parts per million (ppm) relative to the internal standard, tetramethylsilane. Coupling constants (*J* values) are reported in hertz. LC-MS and electrospray mass spectra were recorded in positive or negative mode (Quattro microTM, Waters, Milford, MA; 2795 Separations Module, 2996 Photodiode Array Detector). The sample was applied to C18 column (XBrigeTM, Waters, 2.1 x 50 mm, 5 µm) with a C18 guard cartridge (XBrigeTM, Waters, 2.1 × 10 mm, 5 µm). The sample was eluted with water and 0.1% AcOH buffer (solvent A) and acetonitrile (solvent B) at 1 mL/min with a 4 min gradient (10 % B to 90% B), and maintaining the final conditions for 1 min. Purification was performed by flash column chromatography (FLASH 40+, Biotage M or SNAP KP-SIL cartridges) and by preparative TLC with Kiesel gel 60 F254 (Merck, 1.0 mm). TLC was performed on Kiesel gel 60 F254 (Merck, 0.25 mm) precoated glass plates. Reagents were used without further purification.

**1-Phenyl-2,5,8,11-tetraoxatridecan-13-yl toluenesulfonate(2)**

*p*-Toluenesulfonyl chloride (1.28 g, 6.71 mmol) was slowly added to a solution of commercially available tetraethyleneglycol monobenzyl ether **1** (2.00 mL, 7.74 mmol) in triethylamine (1.78 mL, 12.8 mmol) and dichloromethane (25 mL) on ice. The mixture was stirred overnight at room temperature. Brine was added and the mixture was extracted with dichloromethane. The organic layer was dried over anhydrous sodium sulfate and evaporated. Flash chromatography of the residue over silica gel (elution: hexane/ethyl acetate 4:1 to 2:1) provided **2** (1.65 g, 3.77 mmol) as a colorless oil in 49% yield. ES-MS : 439 (M + H)^+^.

**13-Azido-1-phenyl-2,5,8,11-tetraoxatridecane(3)**

A mixture of 1-phenyl-2, 5, 8, 11-tetraoxatridecan-13-yl toluenesulfonate **2** (1.65 g, 3.77 mmol) and sodium azide (293 mg, 4.51 mmol) in dimethylformamide (10 mL) was stirred overnight at room temperature. The reaction mixture was poured into brine and extracted with dichloromethane. The organic layer was dried over anhydrous sodium sulfate and evaporated to provide crude **3**. This material was used for the next reaction without further purification. ES-MS : 310 (M + H)^+^.

**1-Phenyl-2, 5, 8, 11-tetraoxatridecan-13-amine (4)**

Triphenylphosphine (592 mg, 2.26 mmol) and H_2_O (1 mL) were added to a solution of half of the crude azide **3** in THF (3 mL). After being stirred for 4 h at room temperature, concentration of the mixture provided crude **4**. This material was used for the next reaction without further purification. ES-MS: 284 (M + H)^+^.

**4-[(3a*S*,4*S*,6a*R*)-2-Oxohexahydro-1H-thieno[3,4-*d*]imidazol-4-yl]-*N*-(1-phenyl-2,5,8,11-tetraoxatridecan-13-yl)butanamide (5)**

(+)-Biotin (551 mg, 2.26 mmol), HBTU (856 mg, 2.26 mmol) and *N*,*N*-ethyldiisopropylethylamine (393 µL, 2.28 mmol) was added to a solution of crude amine **4** in dichloromethane (4 mL). The mixture was stirred overnight at room temperature. Brine was added and the mixture was extracted with dichloromethane. The organic layer was washed with saturated aqueous NaHCO_3_ solution, dried over anhydrous sodium sulfate, and evaporated. Flash chromatography of the residue over silica gel (CHCl_3_/MeOH 100:3) provided **5** (454.0 mg, 0.89 mmol) as a white solid in 24 % yield over three steps. ^1^H NMR (500 MHz, CDCl_3_) δ: 7.35–7.28 (5H, m), 6.91 (1H, t, *J* = 5.7 Hz), 6.41 (1H, brs), 5.45 (1H, brs), 4.57 (2H, s), 4.48 (1H, dd, *J* = 12.5 Hz, 7.4 Hz), 4.28 (1H, m), 3.69–3.61 (11H, m), 3.56 (2H, t, *J* = 12.5 Hz, 7.4 Hz), 3.43 (2H, m), 3.11 (1H, m), 2.89 (1H, dd, *J* = 12.5 Hz, 5.1 Hz), 2.73 (1H, d, *J* = 12.5 Hz), 2.22 (2H, m), 2.06 (2H, m), 1.78–1.61 (3H, m), 1.42 (2H, m). ES-MS (*m/z*): 510 (M + H)^+^.

**13-Oxo-16-[(3a*S*,4*S*,6a*R*)-2-oxohexahydro-1H-thieno[3,4-*d*]imidazol-4-yl]-3,6,9-trioxa-12-azahexadecyl 4-{5-[bis(2-chloroethyl)amino]-1-methyl-1H-benzo[*d*]imidazol-2- yl}butanoate (6)**

A mixture of **5** (33.1 mg, 0.07 mmol) and 10% Pd-C (50% wet, 50 mg) in AcOH (5 mL) and H_2_O (1 mL) was hydrogenated for 7 h under H_2_ at room temperature. After the catalyst was removed with celite, evaporation of the solution provided the crude hydrogenated product **5** (31.9mg) as a brown oil. ES-MS: 420 (M + H)^+^.

BENDA HCl (12.8 mg, 0.03 mmol), *N*,*N*’-diisopropylcarbodiimide (5 µL, 0.03 mmol) and 4-dimethylaminopyridine (0.4 mg, 0.003 mmol) were added to a solution of the crude hydrogenated product **5** (27.2 mg) in dichloromethane (2 mL) at 0 °C under Ar atmosphere. After being stirred at 0 °C for 1 h, 4-dimethylaminopyridine (0.4 mg, 0.03 mmol) was added and the reaction mixture was stirred for 1h at 0 °C. After further addition of *N*,*N*’-diisopropylcarbodiimide (10 µL, 0.06 mmol), the mixture was stirred for 1 h at room temperature. The reaction mixture was poured into ice-cooled saturated aqueous NaHCO_3_, extracted with dichloromethane, dried over anhydrous sodium sulfate, and evaporated. The crude material was purified by preparative TLC (CHCl_3_/MeOH = 10/1) to provide the titled molecule **6** (16.1 mg, 0.02 mmol) as a brown oil in 29 % yield over two steps. ^1^H NMR (500 MHz, CDCl_3_) δ: 7.19 (1H, d, *J* = 9.1 Hz), 7.08 (1H, d, *J* = 2.3 Hz), 6.79 (1H, dd, *J* = 9.1 Hz, 2.3 Hz), 6.67 (1H, br), 6.31 (1H, brs), 5.39 (1H, brs), 4.50 (1H, m), 4.31 (1H, m), 4.23 (2H, t, *J* = 5.1 Hz), 3.75-3.41 (23H, m), 3.39 (2H, t, *J* = 5.1 Hz), 3.14 (1H, m), 2.94-2.89 (3H, m), 2.74 (1H, d, *J* = 12.5 Hz), 2.54 (2H, t, *J* = 6.8 Hz), 2.26-2.17 (4H, m), 1.77-1.64 (4H, m), 1.45 (2H, m). ES-MS (*m/z*): 763 (^37^Cl^37^ClM + H)^+^, 761 (^37^Cl ^35^Cl M + H)^+^, 759 (^35^Cl ^35^Cl M + H)^+^.
